# Effect of Dextran‐Aminated Hyaluronic Acid Hydrogel on the Repair of Different Degrees of Epidermal Injury

**DOI:** 10.1111/jocd.70594

**Published:** 2025-12-13

**Authors:** Yao Liu, Chenyu Liu, Zilin Zhang, Tong He, Shiwei Wang, Weihong Qiao, Lin Wang

**Affiliations:** ^1^ Plastic and Cosmetic Surgery Department Central Hospital of Dalian University of Technology Dalian Liaoning China; ^2^ School of Chemical Engineering State Key Laboratory of Fine Chemicals, Dalian University of Technology Dalian Liaoning China; ^3^ Bejing Engineering Lab of Neo‐Biodegradable Materials Beijing China

**Keywords:** chemical peel, dextran, epidermal, medical cosmetology, repair

## Abstract

**Background:**

Postskin peeling procedures commonly result in epidermal injuries with the potential for infection and suboptimal aesthetic outcomes. In clinical practice, dextran and hyaluronic acid (HA) are frequently used as adjunctive materials to enhance wound healing.

**Aims:**

To evaluate the efficacy of a dextran‐aminated hyaluronic acid (DA‐AHA) hydrogel in epidermal injury repair.

**Methods:**

Murine models of stratum corneum and epidermal layer injury were established. Nine mice with disrupted stratum corneum barriers and 45 with disrupted epidermal barriers were randomized into three groups: Saline, HA gel, and DA‐AHA. The repair efficacy of the DA‐AHA gel was assessed by measuring the trans epidermal water loss (TEWL) at the injury site and conducting skin tissue pathological examinations.

**Results:**

DA‐AHA gel more rapidly reduced TEWL at the injury site compared with both saline and HA gel, leading to accelerated wound repair. Pathological and fluorescence staining analyses indicated that DA‐AHA gel could form a dense film at the wound site, subsequently reducing inflammatory cell infiltration and tumor necrosis factor‐α expression while increasing transforming growth factor‐β1 levels. Consequently keratinocyte proliferation, migration, and differentiation were enhanced. Additionally, DA‐AHA gel induced the thickening of the epidermal layer during the later stages of repair.

**Conclusions:**

DA‐AHA gel can expedite the repair of injured epidermis, alleviate inflammation of the affected area, and thicken the epidermal layer. Therefore, DA‐AHA gel is an efficacious treatment in epidermal injury repair, with potential clinical application.

## 1 Introduction

Skin resurfacing techniques, including laser resurfacing, chemical peels, microdermabrasion, and microneedling [1, 2, 3, 4], are widely used to treat skin conditions such as acne and vitiligo, as well as in medical cosmetology [5, 6]. In particular, chemical peels have become one of the most popular nonsurgical cosmetic procedures [6, 7]. Chemical peels selectively remove the epidermis or stratum corneum and stimulate epidermis regeneration and repair, thereby rejuvenating aged or compromised skin, improving skin elasticity, and creating a youthful appearance [1, 8, 9, 10].

The restoration of damaged skin is crucial for ensuring the efficacy of nonsurgical cosmetic procedures [11, 12]. Inadequate postprocedural care can lead to inflammation, hyperpigmentation, delayed healing, and scarring [13, 14]. Barrier disruption of the skin can also accelerate moisture loss and increase the risk of pathogen invasion [15, 16]. Dressings are generally recommended after peeling, along with sun protection, moisturization, and the use of anti‐infective medications [11, 17]. Gel dressings, due to their excellent biocompatibility, adjustable mechanical properties, moisture retention capabilities, and antimicrobial attributes, have become one of the most commonly used postprocedural care products [18, 19]. Hyaluronic acid (HA) and dextran are frequently used in the preparation of gel dressings [20, 21, 22, 23]. However, gels prepared using HA or glucans alone exhibit suboptimal mechanical properties, which can be improved through physical or chemical modifications [24, 25].

In a previous study, modified dextran was combined with HA to fabricate a dextran‐aminated hyaluronic acid (DA‐AHA) gel, which demonstrated enhanced full‐thickness skin wound repair efficacy [26]. In this study, we established murine stratum corneum and epidermal wound models using the tape‐stripping method to simulate varying degrees of epidermal injury after peeling. The efficacy of DA‐AHA gel in promoting healing was compared with that of commercial repair gels.

## 2 Materials and Methods

### 2.1 Materials

DA‐AHA gel was prepared and freeze‐dried following established methods [26]. The freeze‐dried fibers were dissolved in normal saline to a concentration of 100 mg/mL and mixed to form a gel, which was freshly prepared prior to each use. HA gel was procured from Sunho Bioengineering Co. Ltd. (Guangzhou, China); medical tape was obtained from Hainuo Biological Engineering Co. Ltd. (Qingdao, China); and all antibodies were purchased from Servicebio Technology Co. Ltd. (Wuhan, China).

### 2.2 Animals and Models

#### 2.2.1 Experimental Animals

Fifty‐four male, 8‐week‐old Kunming mice were provided by SPF Biotechnology Co. Ltd. (Beijing, China). All the animals were housed under comfortable conditions with ad libitum access to food and water.

#### 2.2.2 Stratum Corneum Barrier Disruption Model

Nine mice were anesthetized with isoflurane, and the fur on the back was shaved. Skin barrier disruption was induced using the classic tape‐stripping method [27, 28, 29]; the tape was cut into 1.2 × 1.5 cm^2^ strips and applied laterally to the back of the mice, followed by rapid removal. Trans epidermal water loss (TEWL) was measured before and after injury using a skin elasticity tester (Cutometer Dual MPA580, Courage + Khazaka Electronic GmbH, Germany). The model was considered to have been established when TEWL increased by approximately 7 g/m^2^/h.

#### 2.2.3 Epidermal Barrier Disruption Model

**FIGURE 1:**
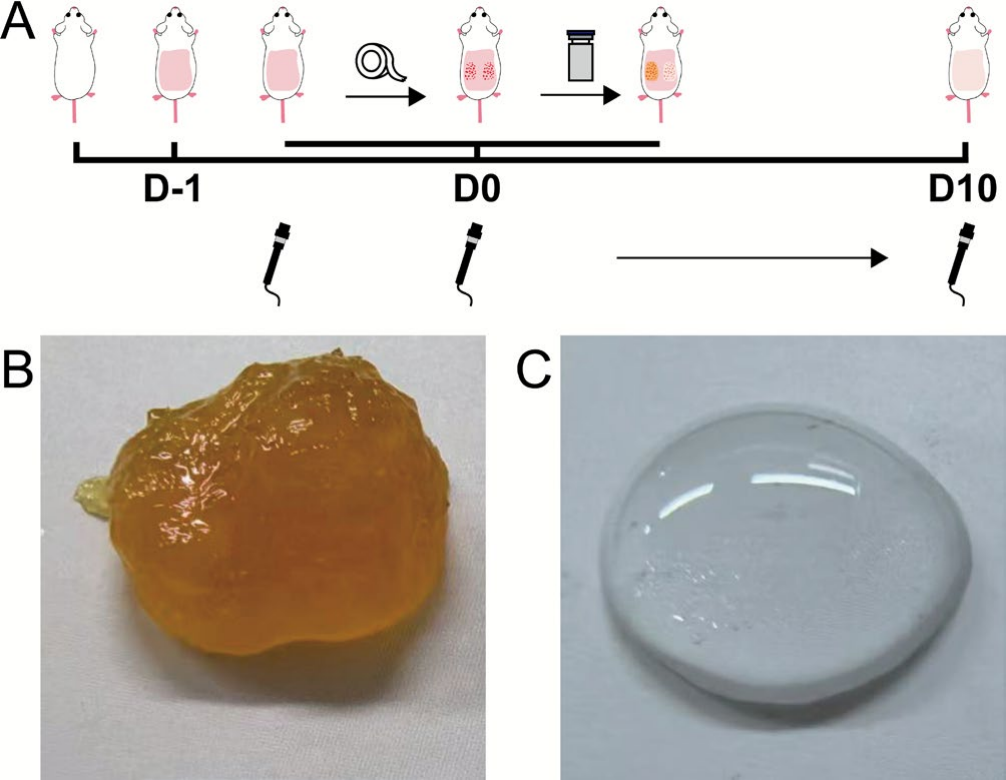
Study design and gel morphology. (A) Experimental procedure of epidermal barrier injury. On the day of the test, the epidermal layer of the mice's skin was stripped using tape to create an injury, and the samples (saline, HA‐gel, or DA‐AHA gel) were then evenly applied to the injury site. TEWL was measured both before and after inducing the injury. Subsequently, TEWL measurements were taken daily, and application of the samples was repeated daily until day 10 (D10). Morphology of (B) the DA‐AHA gel and (C) the HA‐gel. DA‐AHA, dextran‐aminated hyaluronic acid; HA, hyaluronic acid; TEWL, transepidermal water loss.

Epidermal layer damage was induced in 45 mice following the procedure outlined in Section 2.2.2. Tape stripping was repeated 30–50 times until significant skin damage was observed. TEWL was measured and recorded before and after stripping. The establishment of the model and the treatment process is depicted in Figure 1A.

### 2.3 Treatment and Evaluation

Mice with disrupted stratum corneum barriers (*n* = 9, Section 2.2.2) were randomized into three groups: Saline, HA gel, and DA‐AHA. Each mouse had two injured dorsal sites (left/right), totaling 18 evenly randomized sites (six per group). The samples—saline, HA gel, and DA‐AHA—were gently applied to cover their respective injured sites: No additional treatment was performed (Figure 1B,C). Three noninjured, untreated dorsal locations across the cohort served as the control group (normal skin). TEWL was measured exclusively at the injured sites every 3 h for 24 h. After the final measurement, the mice were euthanized and injured/normal skin samples were excised for histopathology.

For mice with disrupted epidermal barriers (*n* = 45, Section 2.2.3), treatment was performed as described above: mice were randomized into Saline, HA gel, and DA‐AHA groups, with each mouse having two injured dorsal sites (left/right), totaling 90 evenly randomized injured sites (30 per group). The corresponding samples—saline, HA‐gel, and DA‐AHA—were gently applied once daily for 10 consecutive days to cover their respective injured sites. TEWL was measured exclusively at the injured sites at a fixed time daily. At the end of the measurements on Day 0 (D0), D1, D3, D7, and D10, nine mice were euthanized at each time point (to ensure that each group had six injured sites); injured skin (from treated sites) and normal skin (from noninjured blank control sites, as referenced earlier) were excised for pathological examination.

### 2.4 Histopathological Staining

The excised mouse skin was fixed in neutral formalin, embedded in paraffin wax, and prepared into 5‐μm sections. The sections were stained with hematoxylin and eosin (H&E) using standard protocols. The stained sections were observed using image capture software [Aperio ImageScope 12.4.6, Leica Microsystems (Shanghai) Trading Co. Ltd., Shanghai, China], and the thickness of the epidermis was measured.

### 2.5 Immunofluorescence Staining

The sections were subjected to immunofluorescence staining following the manufacturer's standard protocols to observe the expression levels of tumor necrosis factor (TNF)‐α and transforming growth factor (TGF)‐β1 in the damaged skin, as well as the keratins K1 and K14 associated with keratinocyte proliferation and differentiation. K1 labels terminally differentiated keratinocytes (mainly in the upper epidermis), indicating the fully differentiated cells with involvement in epidermal barrier repair; K14 labels undifferentiated keratinocytes (primarily in the epidermal basal layer), representing the cells with strong proliferative and migratory capacity critical for initiating epithelial repair [30, 31, 32, 33]. Fluorescence staining results were observed using image capture software (Slide Viewer 2.5.0; 3DHISTECH, Budapest, Hungary).

### 2.6 Data and Statistical Analyses

Data processing, statistical analyses, and graphic creation were performed using GraphPad Prism version 8 (GraphPad Software, San Diego, CA, USA). Differences among multiple groups were evaluated using one‐way analysis of variance (ANOVA), and Tukey's multiple comparison test was used for pairwise comparisons. Statistical significance was set at *p* < 0.05. All data are expressed as mean ± standard deviation (SD).

## 3 Results

### 3.1 Stratum Corneum Barrier Injury

#### 3.1.1 TEWL

**FIGURE 2:**
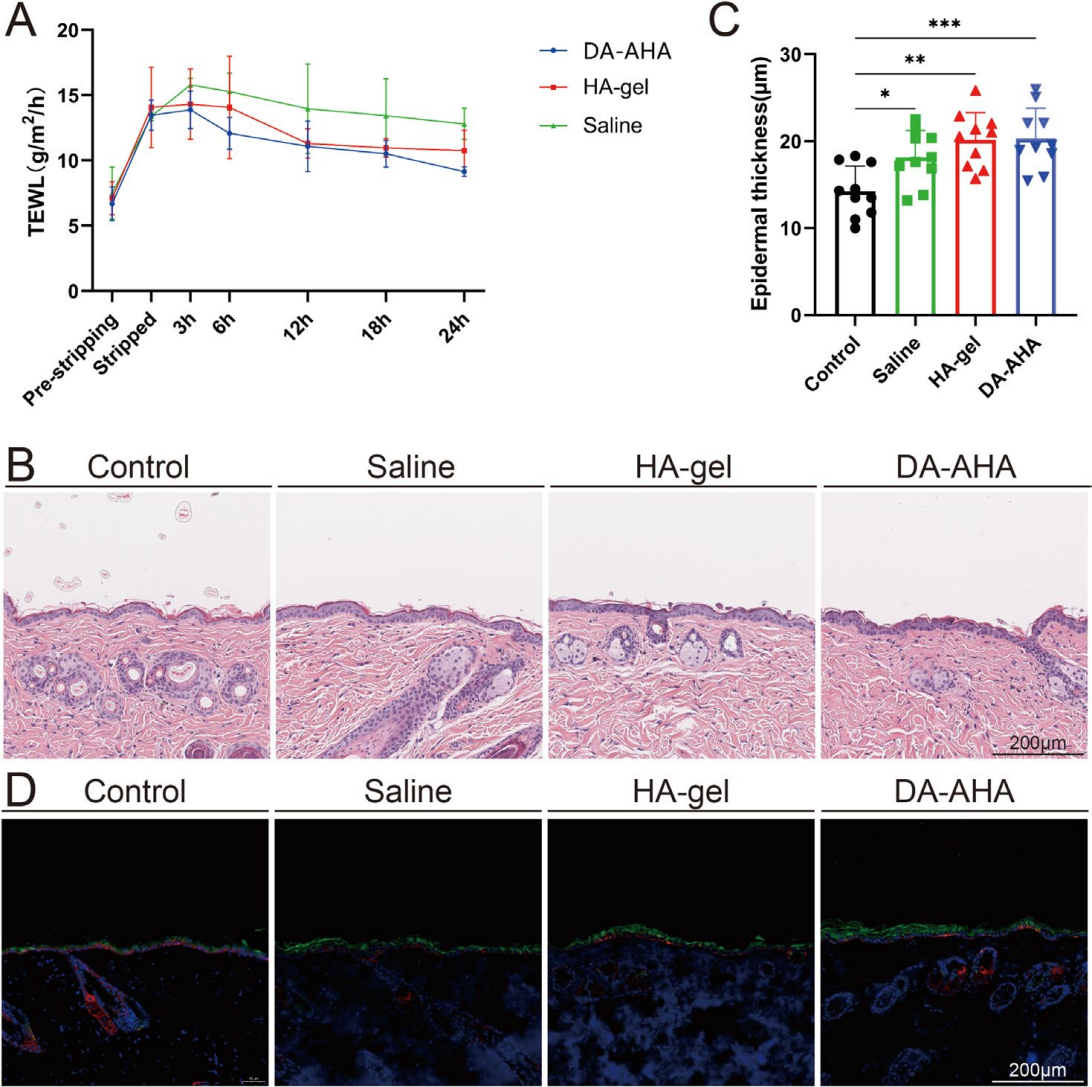
Evaluation of the repair effect in mice with stratum corneum barrier injury. (A) TEWL at the injured site. TEWL at the injured site was measured at various time points before and after inducing the injury. (B) Pathological examination results of the injured site and (C) epidermal thickness. At 24 h after treatment with different samples, the skin at the injured site was excised and stained with H&E. The epidermal thickness was then measured. (D) Immunofluorescence staining of the injured site after treatment. Immunofluorescence staining was performed to visualize the expression of K1 (green), K14 (red), and nuclei (DAPI, blue) at the injured site (scale bar: 200 μm). H&E, hematoxylin and eosin; K1, keratin 1; K14, keratin; 14; TEWL, transepidermal water loss.

After tape stripping the stratum corneum, the TEWL at the injured site significantly increased from the noninjured level of 7.13 ± 1.55 g/m^2^/h to 13.60 ± 1.42 g/m^2^/h. After applying the sample (saline, HA‐gel, or DA‐AHA) for 3 h the TEWL of the HA‐gel and DA‐AHA groups was lower than that of the saline group. After 6 h, the DA‐AHA group exhibited the lowest TEWL. Within 24 h, the TEWL at the injured sites decreased in all groups, with the DA‐AHA group exhibiting the greatest reduction (9.16 ± 0.37 g/m^2^/h), followed by the HA‐gel group (10.76 ± 1.56 g/m^2^/h) and the saline group (12.81 ± 1.20 g/m^2^/h) (Figure 2A).

#### 3.1.2 H&E and Immunofluorescence Staining

Histopathological analysis revealed that 24 h after tape stripping all groups displayed thickening of the epidermal layer compared with the nonstripped skin, although no significant differences were observed between the groups (Figures 2B,C). Immunofluorescence staining indicated an increase in the number of K1‐positive cells within the epidermal layer in all groups after tape stripping (Figures 2D,S1A).

### 3.2 Epidermal Barrier Injury

#### 3.2.1 TEWL

**FIGURE 3:**
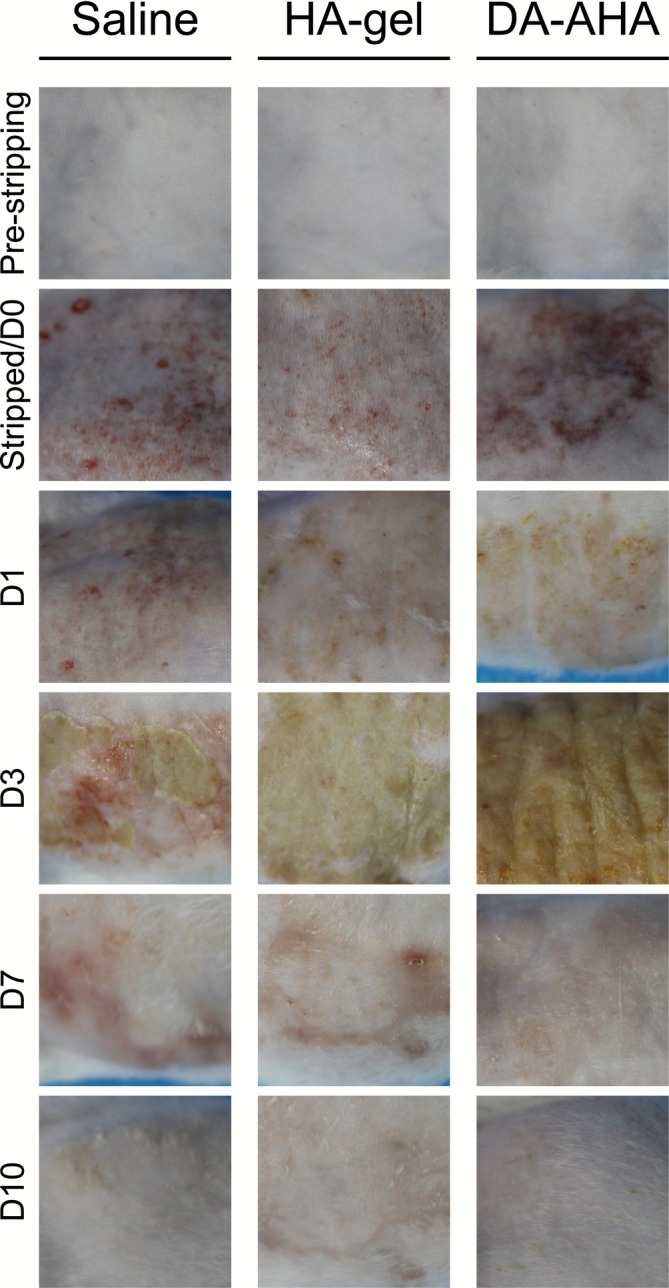
Wound repair process in mice with epidermal barrier injury. Throughout the experiment, the skin at the injured site was photographed daily at a fixed time to monitor the progression of wound repair.

**FIGURE 4:**
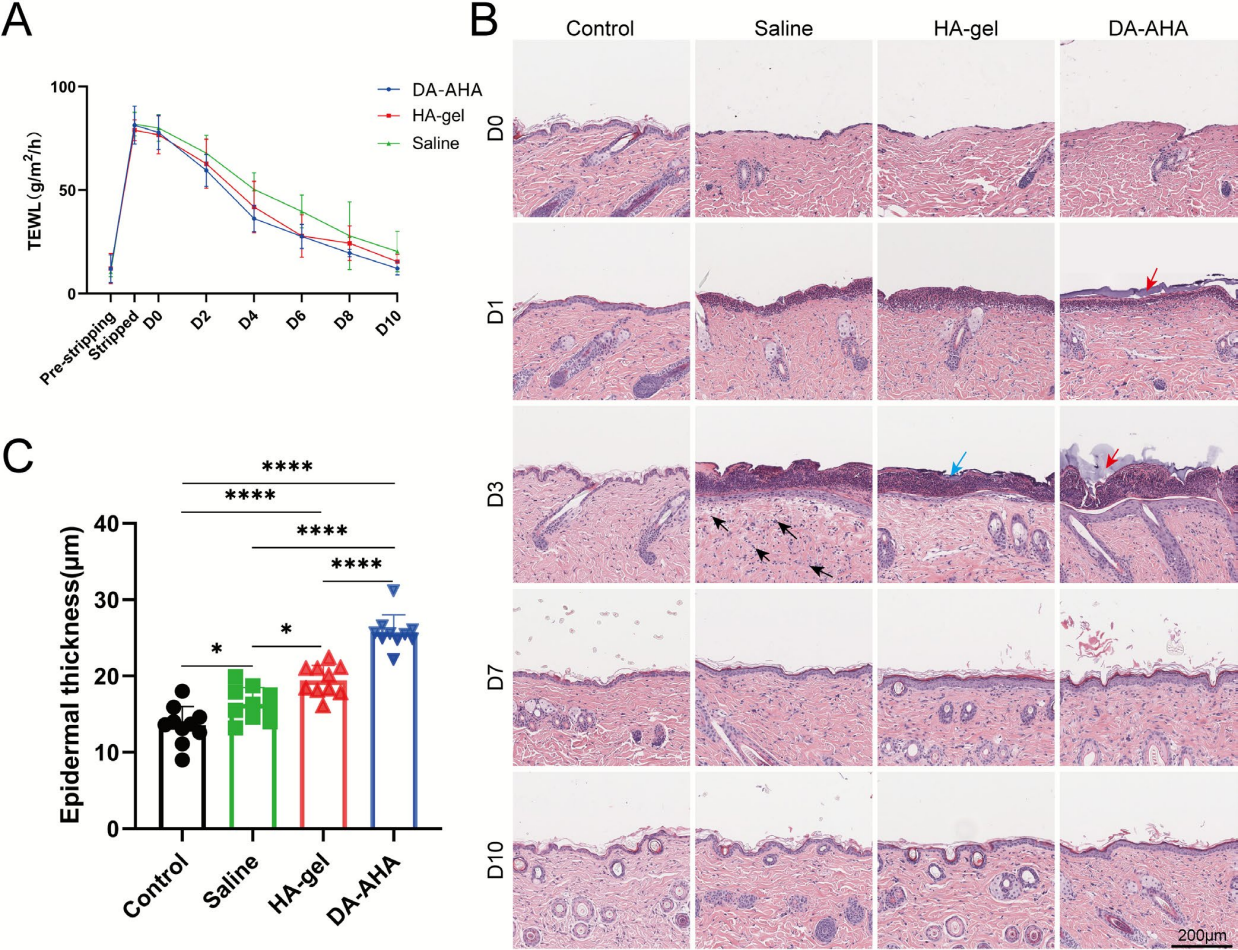
Evaluation of the repair effect in mice with epidermal barrier injury. (A) TEWL at the injury site. TEWL at the site of injury was measured at various time points before and after the injury to assess barrier recovery. (B) Pathological examination results of the injured site and (C) epidermal thickness. At different time intervals following treatment with various samples, the skin at the injury site was excised and stained with H&E. The epidermal thickness was then measured to evaluate the repair efficacy (scale bar: 200 μm, black arrows: Inflammatory cells, red arrows: DA‐AHA gel, blue arrows: Necrotic cells). DA‐AHA, dextran‐aminated hyaluronic acid; H&E, hematoxylin and eosin; TEWL, transepidermal water loss.

The TEWL of the noneroded mouse skin was 11.52 ± 5.77 g/m^2^/h. Regarding the discrepancy in baseline TEWL values between the two models, we speculate it may be attributed to batch‐specific differences in mice or fluctuations in the laboratory's relative humidity. Continuous tape stripping resulted in pronounced skin damage (Figure 3). Concurrently, the TEWL at the injured site dramatically increased to 80.75 ± 6.42 g/m^2^/h (Figure 4A). Following 10 days of treatment, TEWL values at the injured sites significantly decreased in all groups; the DA‐AHA group showed the most substantial reduction (12.22 ± 3.16 g/m^2^/h), followed by the HA‐gel group (15.48 ± 3.48 g/m^2^/h) and the Saline group (20.40 ± 9.81 g/m^2^/h).

#### 3.2.2 Injury Repair

From D0 to D3, all groups exhibited scab formation and skin contraction. Notably, the DA‐AHA group showed more extensive scab formation and skin contraction compared with the Saline and HA‐gel groups. This was attributed to the dense film formed by DA‐AHA gel at the wound site serving as a physical protective barrier (consistent with subsequent pathological observations), rather than to aggravated tissue damage. By D7, scab shedding had occurred in all the groups, with the DA‐AHA group exhibiting faster scab detachment compared with the Saline and HA‐gel groups, indicating accelerated repair. By D10, the healing process was predominantly complete, with the DA‐AHA group demonstrating no scarring, the HA‐gel and Saline groups exhibiting slight scarring, and new hair growth observed in all the groups (Figure 3).

#### 3.2.3 H&E and Immunofluorescence Staining

Histopathological analysis showed that all the groups exhibited epidermal stripping immediately after injury(Figure 4B). From D1 to D3, necrotic cells were abundant on the skin surface at the injured site, initiating scab formation. On D3, the Saline group presented with the highest number of inflammatory cells in the dermis, and the DA‐AHA group had the fewest. By D10, all groups demonstrated complete healing, with an orderly arrangement of epidermal cells. Compared with the undamaged skin, the damaged epidermis in all groups exhibited thickening (Figure 4C), with the DA‐AHA group showing the most significant thickening and a denser stratum corneum.

**FIGURE 5:**
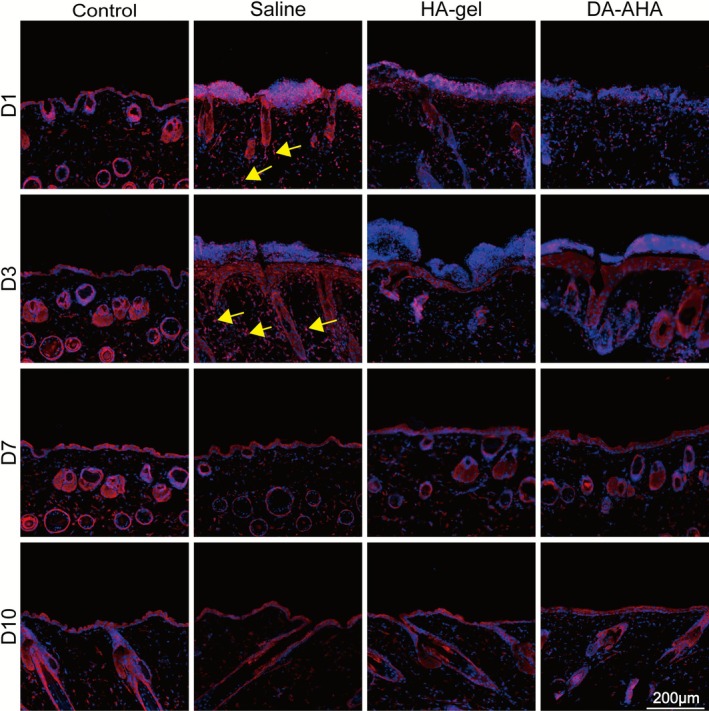
Immunofluorescence staining of TNF‐α expression at the injured site. TNF‐α expression patterns were assessed through immunofluorescence staining at various post‐treatment time points in the animal model. Yellow arrows indicate cells demonstrating elevated TNF‐α expression. TNF‐α (red), nuclei (DAPI, blue) (scale bar: 200 μm). TNF‐α, tumor necrosis factor‐alpha.

TNF‐α fluorescence staining (Figures 5, S1B) revealed that the expression level of TNF‐α increased in the Saline group from D1 to D3, with a significant number of TNF‐α‐positive cells visible in the dermis. The TNF‐α levels gradually decreased from D1 in the HA‐gel and DA‐AHA groups, with the DA‐AHA group having the fewest TNF‐α‐positive cells.

**FIGURE 6:**
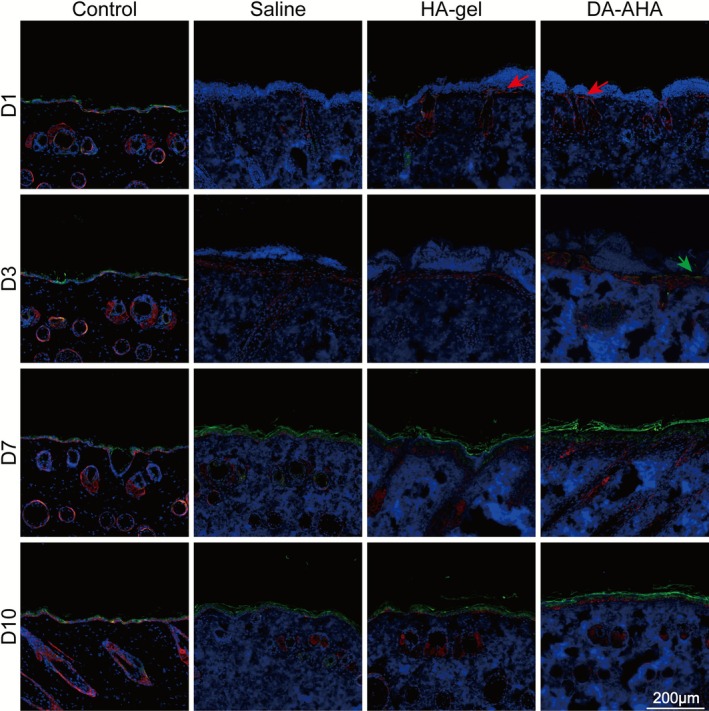
Immunofluorescence staining of K1 and K14 expression at the injured site. Immunofluorescence staining of K1 and K14 at various time points after treating mice with epidermal barrier injury. Red arrows denote keratinocytes migrating toward the injured site and while green arrows indicate keratinized keratinocytes. K1 (green), K14 (red), and nuclei (DAPI, blue) (scale bar: 200 μm). K1, keratin 1; K14, keratin 14.

K1 and K14 were labeled using fluorescent antibodies (Figures 6, S1C). On D1, K14‐positive cells were visible within the hair follicles in all groups, with the DA‐AHA and HA‐gel groups showing red fluorescence signals in the basal layer of the epidermis at the injury site. By D3, a large number of K14‐positive cells were observed covering the basal layer of the epidermis, along with a few K1‐positive cells in the DA‐AHA group. From D7 to D10, the number of K14‐positive cells within the epidermis decreased, whereas the number of K1‐positive cells increased, and a strong K1 green fluorescence signal was present on the skin surface, particularly in the DA‐AHA group. These findings indicate that compared with the other two groups, the DA‐AHA group effectively recruited undifferentiated keratinocytes (from hair follicles and from the basal layer) to the wound site via K14 expression in the early stages of healing, and that these cells migrated to cover the injured area. Subsequently, the significant increase in K1 expression guided keratinocytes toward terminal differentiation, contributing to the formation of a dense stratum corneum.

**FIGURE 7:**
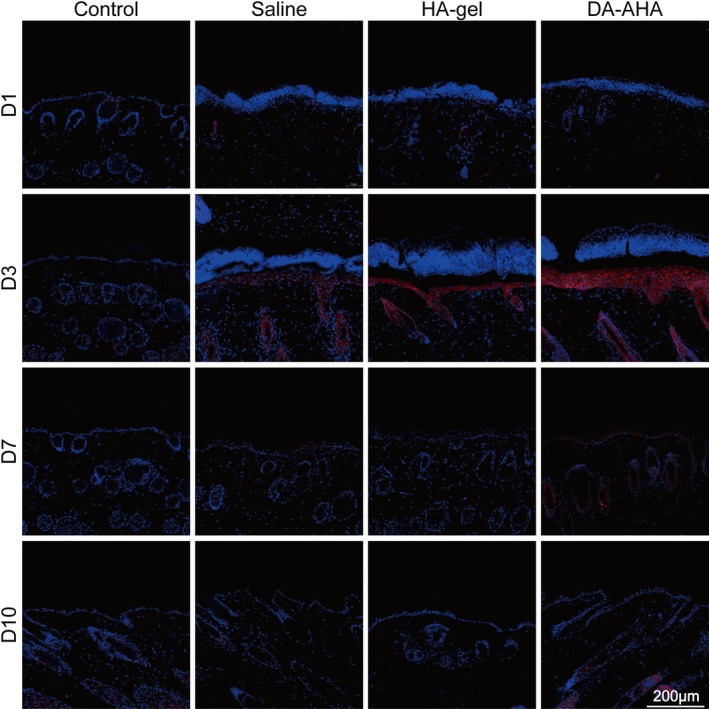
Immunofluorescence staining of TGF‐β1 expression at the injury site. Immunofluorescence staining of TGF‐β1 expression was conducted at various time points after treatment in an experimental model of epidermal barrier injury. TGF‐β1 (red), nuclei (DAPI, blue) (scale bar: 200 μm). TGF‐β1, transforming growth factor‐beta 1.

On D1, TGF‐β1 expression levels were low in all the groups (Figures 7, S1D). On D3, the expression levels increased to their highest levels, predominantly within the new epidermis. The DA‐AHA group had the highest TGF‐β1 expression level, followed by the HA‐gel group and the Saline group. From D7 to D10, the TGF‐β1 expression levels slightly decreased, with the level of the DA‐AHA group slightly higher than that of the other groups.

## 4 Discussion

Our experimental results indicated that DA‐AHA gel effectively reduced TEWL at the site of skin injury, mitigated inflammation, accelerated the healing process of the epidermal layer, and increased the thickness of the epidermis. The postdamage application of DA‐AHA gel to the stratum corneum facilitated a more rapid reduction in TEWL at the injured site compared with the application of saline or HA‐gel. Within 24 h after injury, epidermal cells in the damaged area exhibited proliferation and differentiation, resulting in slight thickening of the epidermis. Daily application of DA‐AHA gel after tape stripping‐induced epidermal damage accelerated the healing process, outperforming the commercially available products. DA‐AHA gel also accelerated scab formation and reduced moisture loss at the wound site. Pathological analysis indicated that DA‐AHA gel can reduce the inflammation at the injured site, enhanced the proliferation and differentiation of epidermal cells, and increased the thickness of the epidermis, resulting in a denser stratum corneum. Immunofluorescence staining confirmed that DA‐AHA gel can decrease the inflammatory response at the injury site and increased the expression of the repair factor TGF‐β1. In addition, DA‐AHA gel promoted keratinocyte proliferation and differentiation, thereby accelerating injury repair.

Skin resurfacing is a common technique used to mitigate skin aging, photoaging, and dermatological conditions. Its core principle involves inducing skin damage, and the subsequent healing process determines the final outcome. Inadequate postpeeling care can lead to skin dryness, erythema, and increased risk of infection. A faster healing rate is often desired and is a key focus in the field of wound repair. In the present study, we assessed the efficacy of DA‐AHA gel in repairing the damaged epidermis.

The epidermis—a barrier between the human body and the external environment—effectively prevents internal moisture loss [34]. We observed that TEWL at the wound site significantly increased under conditions of stratum corneum or epidermal damage. Following treatment, TEWL decreased in all groups, with the level in the DA‐AHA group being lower than that in the HA‐gel and Saline groups, albeit the difference did not reach statistical significance. This can be attributed to the rapid repair capacity of the skin. Notably, organelles in the superficial layer of the skin, such as hair follicles and sweat glands, remain largely undamaged, and this preservation serves as a key contributor to the progression of wound repair [35].

Excessive inflammation can slow the rate of skin repair [36, 37, 38], whereas inhibition of proinflammatory responses during the proliferative phase facilitates wound healing thereby accelerating wound repair [39, 40]. In the saline‐treated group in the current study, the dermis contained a substantial number of neutrophils on D3, whereas the dermis of the HA‐gel group exhibited comparatively fewer granulocytes and macrophages. However, the DA‐AHA group exhibited the least inflammatory cell infiltration into the dermis, and immunofluorescence staining revealed the least infiltration of cells highly expressing TNF‐α. Benefiting from the anti‐inflammatory effects of dextran and HA [41], DA‐AHA gel also reduced the skin levels of TNF‐α at the injured site. The three‐dimensional porous structure of DA‐AHA gel formed a scaffold, promoting cell migration to the wound surface and forming a dense film that covered the wound [42], further blocking pathogen entry and reducing the risk of postoperative infection.

Keratinocytes are essential for skin repair [43]. Following epidermal damage, the surrounding undamaged areas and hair follicles contain epidermal basal cells that proliferate and migrate to the site of injury, differentiate, and form a new epidermal layer. We observed a substantial number of K14‐positive cells within the epidermal layer in the DA‐AHA group on D3, whereas keratinocytes in the differentiation stage exhibited K1 green fluorescence. Specifically, the K14 expression level observed in the DA‐AHA group on D3 confirms that, compared with HA‐gel and saline, DA‐AHA gel mobilizes more undifferentiated keratinocytes to accelerate wound coverage. Its subsequent robust K1 expression (D7–D10) further highlights a key advantage: DA‐AHA gel not only expands keratinocyte numbers but also drives their maturation. Terminally differentiated K1‐positive cells are critical for rebuilding the epidermal barrier directly contributing to the superior TEWL reduction and denser stratum corneum in the DA‐AHA group.

Numerous studies have demonstrated that TGF‐β1 can inhibit keratinocyte proliferation and promote their migration and re‐epithelialization [44, 45, 46]. We evaluated TGF‐β1 expression levels at the injured site and observed higher TGF‐β1 expression within the epidermal layer in the DA‐AHA group on D3. It is hypothesized that this may be attributed to the initial completion of division and proliferation required for repair by basal layer keratinocytes in the DA‐AHA group on D3, leading to increased TGF‐β1 production, which subsequently inhibited keratinocyte proliferation and promoted their differentiation. Notably, while previous studies have noted the role of TGF‐β1 in inhibiting keratinocyte proliferation in the later repair stages, its function is stage‐dependent in early healing: on D3 (when keratinocytes are actively proliferating, as shown by high K14 expression), elevated TGF‐β1 mainly promotes keratinocyte migration to accelerate wound coverage, rather than suppressing proliferation. This overlap between the peak TGF‐β1 level and active proliferation reflects its context‐specific role—supporting early migration while preparing keratinocytes for later differentiation (evident from subsequent K1 upregulation). Additionally, both glucan and HA elevated TGF‐β1 levels at the wound site [47, 48], further inhibiting excessive keratinocyte proliferation and promoting their migration and differentiation toward the epidermis. Given that keratinocyte and fibroblast proliferation and migration are crucial for wound repair—particularly for chronic wound healing [49]—elevated TGF‐β1 levels also contribute to this process by promoting dermal fibroblast proliferation and collagen synthesis [50] Moreover, we observed increased epidermal thickness after repair in both injury models with varying degrees of injury. In particular, we noted significant thickening of the epidermis in the DA‐AHA group in the exfoliation model (Figure 4C), which could lead to enhanced skin barrier function and hydration [51, 52].

The epidermal repair efficacy of DA‐AHA gel was evaluated and compared with that of a commercially available repair gel. Our findings indicated that DA‐AHA gel can accelerate the repair process of the damaged epidermis, reduce water loss and mitigate inflammation at the injured site, and enhance keratinocyte proliferation and migration. Furthermore, as the thickness of the repaired epidermal layer increased, the stratum corneum became more compact, and the overall repair performance of DA‐AHA gel exceeded that of commercial products.

However, there were certain limitations to our study. Previous research has demonstrated a significant impact of DA‐AHA gel on accelerating wound healing [26]. In the current investigation, we focused solely on the repair capabilities of the gel following superficial skin injuries in healthy mice with a low risk of infection. Given the inherent robust regenerative capacity of animal skin and the nonsevere nature of skin injuries [53], the rapid repair effects of the gel were less pronounced, despite observable differences at the histopathological level. In addition, rather than employing laser ablation or chemical peels commonly used in clinical settings, we utilized a more cost‐effective approach of applying adhesive tape to remove the epidermal layer of the skin in mice. Despite achieving the same objectives and ensuring consistent damage through post‐stripping TEWL measurements, the use of alternative methods may introduce unforeseen variations. Moreover, the study design did not incorporate the functional validation of resistance to infection. Future research should involve more skin‐related cell types and adopt more comprehensive molecular biological analyses—such as investigating interactions with the epidermal stem cell‐associated Wnt/β‐catenin signaling pathway [54] or exploring crosstalk between this pathway and others, such as TGF‐β1/Smad, along with key molecular targets—to elucidate the underlying principles of its mechanism of action. The biological activity and mechanisms of action of DA‐AHA gel should be explored in depth in future studies.

## 5 Conclusion

DA‐AHA gel demonstrated heightened reparative efficacy in damaged epidermal tissue. This gel effectively mitigated inflammation at the site of injury, promoted the proliferation and migration of keratinocytes, and accelerated healing and re‐epithelialization of the wound area. Additionally, it concurrently reduced moisture loss from the injured site and enhanced the thickness of the epidermal barrier. Therefore, DA‐AHA gel may be clinically applied as an efficacious treatment in epidermal injury repair.

## Author Contributions

Lin Wang: Conceived of the idea and designed the experiments. Yao Liu and Chenyu Liu with the help of Tong He and Zilin Zhang: Performed the animal experiments and data analysis. Yao Liu and Chenyu Liu with the supervision of Shiwei Wang and Weihong Qiao: Wrote the manuscript. Weihong Qiao: Revised the manuscript. All authors approved the final version of the manuscript.

## Funding

This work was supported by the National Natural Science Foundation of China Grant No. 22378049, the Fundamental Research Funds for the Central Universities [DUT22YG221] and Imeik Technology Development Co. Ltd. IMEIK‐GY‐2024010131.

## Ethics Statement

All the animal experiments were conducted in accordance with the guidelines of the Ethics Committee of Beijing Laboratory Animal Research Center Co. Ltd., and were approved by the aforementioned Ethics Committee (IACUA No. BLARC‐SSYY‐DW/013‐JL/001).

## Consent

Lin Wang hereby authorizes Journal of Cosmetic Dermatology to use the accompanying images of this article for the publication of the paper titled ‘Effect of dextran‐aminated hyaluronic acid hydrogel on the repair of different degrees of epidermal injury’. This article does not involve clinical trials.

## Conflicts of Interest

The authors declare no conflicts of interest.

## Supporting information


**Figure S1:** Immunofluorescence relative expression level at the injury site. (A) Expression of K1 (left) and K14 (right) in mice with stratum corneum barrier injury. (B) Expression of TNF‐α in the dermis of mice with epidermal barrier injury. (C) Expression of K1 (upper) and K14 (lower) in mice with epidermal barrier injury. (D) Expression of TGF‐β1 in the dermis of mice with epidermal barrier injury. K1, keratin 1; K14, keratin 14; TNF‐ α, tumor necrosis factor‐alpha; TGF‐β1, transforming growth factor‐beta 1

## Data Availability

The data that support the findings of this study are available from the corresponding author upon reasonable request.
