# Supplementary material for: Effect of Dextran‐Aminated Hyaluronic Acid Hydrogel on the Repair of Different Degrees of Epidermal Injury
Source: J Cosmet Dermatol. 2025 Dec 13;24(12):e70594. doi: 10.1111/jocd.70594 (PMC12701546; doi:10.1111/jocd.70594)
Supplement: Supplementary file 1 — Figure S1: Immunofluorescence relative expression level at the injury site. (A) Expression of K1 (left) and K14 (right) in mice with stratum corneum barrier injury. (B) Expression of TNF‐α in the dermis of mice with epidermal barrier injury. (C) Expression of K1 (upper) and K14 (lower) in mice with epidermal barrier injury. (D) Expression of TGF‐β1 in the dermis of mice with epidermal barrier injury. K1, keratin 1; K14, keratin 14; TNF‐ α, tumor necrosis factor‐alpha; TGF‐β1, transforming growth factor‐beta 1 [file JOCD-24-e70594-s001.docx]

**Fig S1**


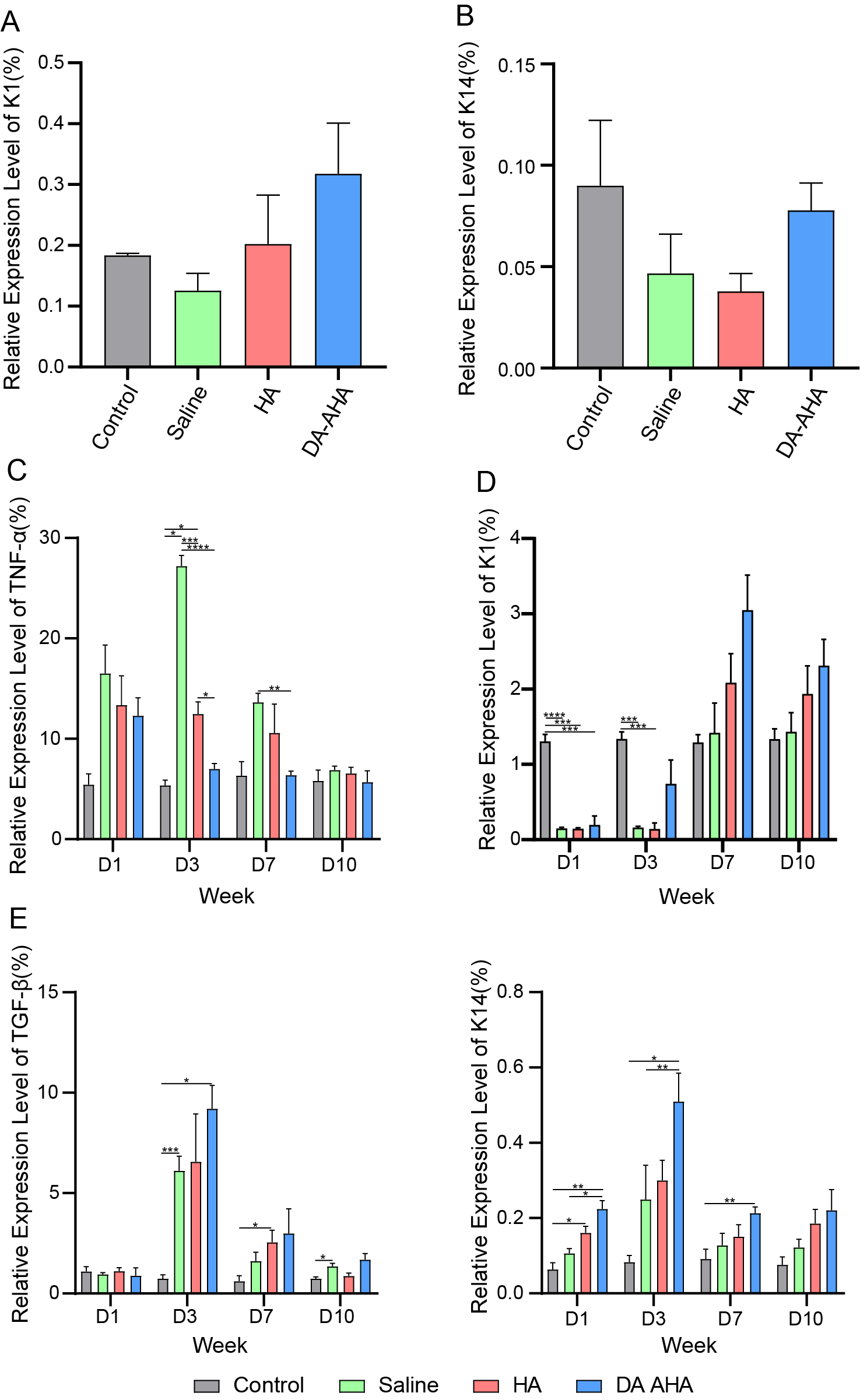


**Figure S1** Immunofluorescence relative expression level at the injury site.

1. Expression of K1 (left) and K14 (right) in mice with stratum corneum barrier injury. (B) Expression of TNF-α in the dermis of mice with epidermal barrier injury. (C) Expression of K1 (upper) and K14 (lower) in mice with epidermal barrier injury. (D) Expression of TGF-β1 in the dermis of mice with epidermal barrier injury.

K1, keratin 1; K14, keratin 14; TNF- α, tumor necrosis factor-alpha; TGF-β1, transforming growth factor-beta 1
